# Taxonomic and Geographic Bias in Conservation Biology Research: A Systematic Review of Wildfowl Demography Studies

**DOI:** 10.1371/journal.pone.0153908

**Published:** 2016-05-11

**Authors:** Beth E. I. Roberts, W. Edwin Harris, Geoff M. Hilton, Stuart J. Marsden

**Affiliations:** 1 Division of Biology and Conservation Biology, School of Science and the Environment, Manchester Metropolitan University, Manchester, United Kingdom; 2 Wildfowl & Wetlands Trust, Slimbridge, Gloucestershire, United Kingdom; Auburn University, UNITED STATES

## Abstract

Demographic data are important to wildlife managers to gauge population health, to allow populations to be utilised sustainably, and to inform conservation efforts. We analysed published demographic data on the world’s wildfowl to examine taxonomic and geographic biases in study, and to identify gaps in knowledge. Wildfowl (order: Anseriformes) are a comparatively well studied bird group which includes 169 species of duck, goose and swan. In all, 1,586 wildfowl research papers published between 1911 and 2010 were found using Web of Knowledge (WoK) and Google Scholar. Over half of the research output involved just 15 species from seven genera. Research output was strongly biased towards ‘high income’ countries, common wildfowl species, and measures of productivity, rather than survival and movement patterns. There were significantly fewer demographic data for the world’s 31 threatened wildfowl species than for non-threatened species. Since 1994, the volume of demographic work on threatened species has increased more than for non-threatened species, but still makes up only 2.7% of total research output. As an aid to research prioritisation, a metric was created to reflect demographic knowledge gaps for each species related to research output for the species, its threat status, and availability of potentially useful surrogate data from congeneric species. According to the metric, the 25 highest priority species include thirteen threatened taxa and nine species each from Asia and South America, and six from Africa.

## Introduction

Biodiversity loss is an international concern [1]. Conservation resources and efforts have helped to prevent some extinctions [2], and yet the rate of biodiversity loss remains at an unparalleled level [3]. The financial resources for conservation are limited, and managers are often required to prioritise which species are targeted for intervention [4]. The IUCN Red List uses information on population size, fragmentation and trend to systematically assign taxa to extinction threat categories [5]. Governments and non-governmental organisations often use the Red List to inform legislation, direct financial investment and prioritise conservation actions [6]. However, using threat status alone for species-based conservation priority approaches may misallocate limited funds [7].

Within conservation science, it is acknowledged that there are biases in our understanding of species’ ecology [8], and that knowledge gaps can fundamentally impede our ability to conserve biodiversity [9]. Both threat status and knowledge gaps are important factors in conservation research priority setting. Although, there are examples of papers that have examined conservation research outputs for biases and knowledge gaps [10], there are no studies that offer a research prioritisation system based on threat and demographic gaps. The current extinction crisis necessitates that the best information be effectively used in population recovery programmes of threatened species [11]. Increasingly, demographic models, such as population viability analysis (PVA) models, which use vital rates to predict extinction risk and population growth rate over time [12, 13], are used to inform management of many species. Demographic information is vital for successful management of species [14]. Population viability analysis models can be used to estimate the probability that a population will decline to a given abundance over a time period, identify the life stage that has the greatest influence on population growth rate, and support assessments of management options to assist in population recovery [15]. Government agencies and conservation bodies are increasingly using PVA models [16]. Thus, there is a need for basic demographic research in species of high conservation priority.

Wildfowl have an almost global distribution [17] and are amongst the most well-studied group of birds due to their importance for hunting, domestication, and aviculture [18]. Nearly one-fifth of wildfowl species are threatened with extinction [19]. Many declining populations are found in Asia and Africa [20], where limited research capacity may result in limited detailed research on species’ demographics. There is a general concern that countries with high levels of biodiversity but low income do not have sufficient resources to study and monitor their important wildlife [21, 22]. Such shortfalls may be the result of a lack of trained biologists [23], of research infrastructure [24], or simply of dedicated finances [25]. Identifying patterns of demographic research volume in relation to taxonomic group, threat status and geographic region is necessary to underpin our goal of increasing the value of future demographic research on the group. This systematic review addresses the following questions: 1. What is the magnitude of geographic and taxonomic bias in demographic research output? 2. How well are we focusing research attention on the world’s threatened wildfowl taxa? 3. Are there disparities in the availability of data on different demographic measures (e.g., productivity, survival, and movement)? 4. How can we devise a single metric which reflects the level of research need for individual species based on its IUCN threat status, the volume of previous work on the species, and availability of data on related species?

## Methods

### Data Sources

**Fig 1:**
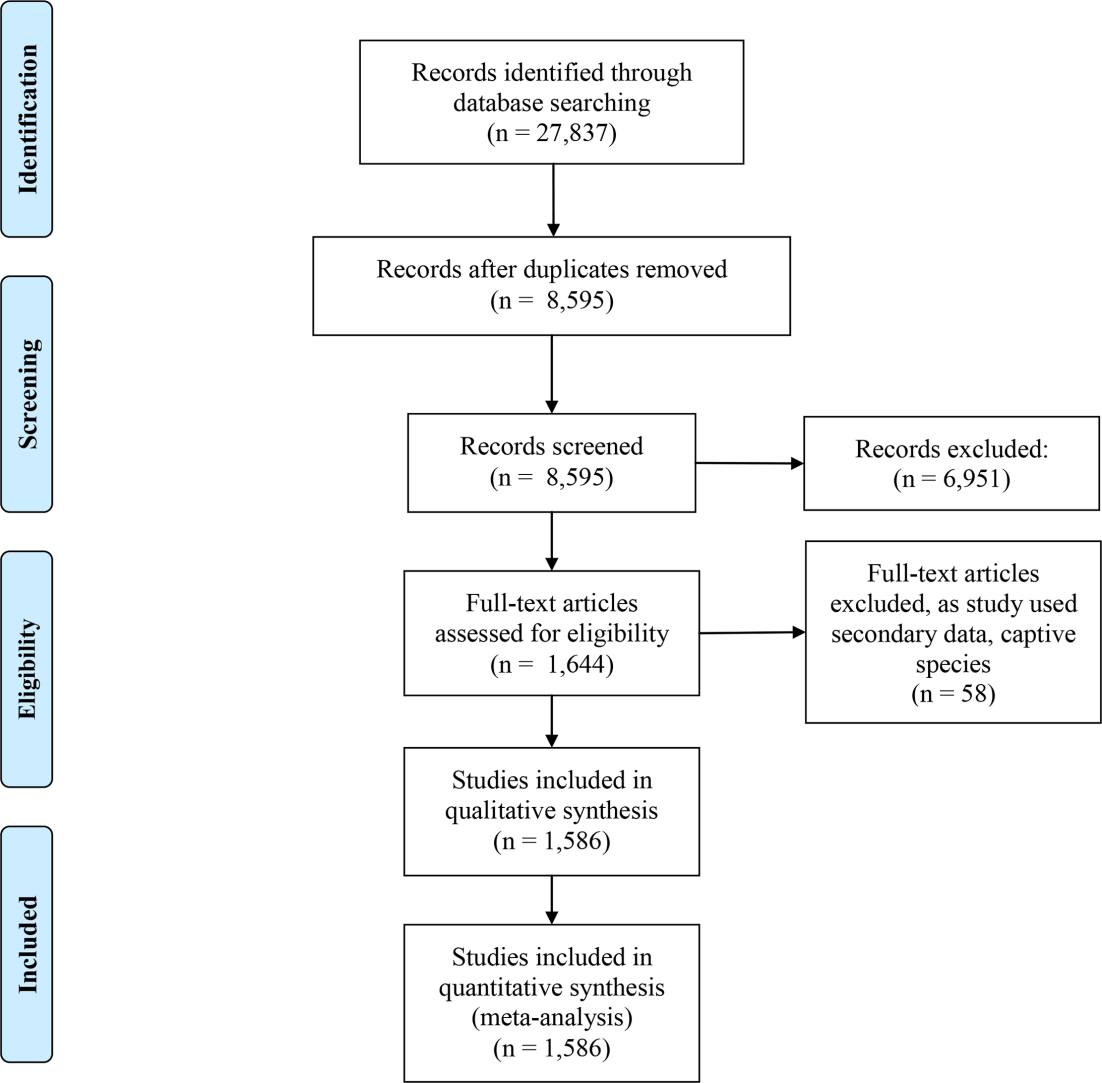
Study selection flow diagram.

Birdlife International has identified 169 extant wildfowl species worldwide, from 55 genera [19]. We conducted a systematic review of the published wildfowl demographic papers using Web of Knowledge (WoK) and Google Scholar between 1 February and 31 August 2012. Reporting followed the Preferred Reporting Items for Systematic Reviews and Meta-Analyses (PRISMA) guidelines. These guidelines include a flow diagram (Fig 1) and a 27-item checklist to ensure good quality systematic reviews [26]. WoK is a worldwide database that contains approximately 23,000 academic and scientific journals [27]. Google Scholar was used particulary to access specialist and regional journals [28], reducing bias in publication selection. Literature within WoK and Google Scholar was searched for by entering, in quotation marks, full scientific and vernacular names and obsolete historic names in the title (S1 Table) from 1911 to 2010. The resulting 8,595 records were screened; the titles and abstracts of all the records were reviewed and all papers that did not include demographic information were excluded. The full text of each paper was scrutinised to determine which demographic variables were reported. Studies of captive birds and those based on secondary data sources were excluded from the analysis. Based upon all the variants of the search terms we identified 1,586 unique papers (Fig 1). For each qualifying paper, we recorded: (1) common and scientific name for each wildfowl species; (2) country; (3) study location; (4) year of publication; and (5) demographic variables presented in the paper. For example, the following were coded from Whitehead et al. (2008) [29]: (1) Blue Duck (*Hymenolaimus malacorhynchos*); (2) New Zealand; (3) Fiord-land National Park; (4) 2008; and (5) sex ratio, clutch size, nest success, duckling survival, fledging success, juvenile survival, adult survival, adult female mortality.

### Data analysis

#### Taxonomic and geographic biases in research output

To aid interpretation of the results, we used World Bank definitions of countries according to their income levels based on 2010 Gross National Income (GNI) per capita (Atlas methodology). These are: ‘high income’ (GNI ˃US$12,476); ‘upper middle income’ (GNI $4,036–12,475); ‘lower middle income’ (GNI $1,026–4,035); ‘low income’ (GNI ˂$1,025) [30]. The distribution of research was mapped using open source mapping software in R [31]. In order to assess the specific effect on research output of GNI and the number of wildfowl, we built a generalised linear model (GLM). We analysed research output as a function of GNI and the number of wildfowl occurring, using a GLM with a negative binomial error structure, following the methods of Zuur et al. 2009 [32].

#### Biases in demographic research

Parameters recorded in the published papers were grouped into three demographic measures: (1) productivity; (2) survival; and (3) movement. The productivity category included age ratio, sex ratio, clutch size, breeding propensity, nest success (the probability of one or more eggs hatching), hatching success (the proportion of eggs in a successful clutch that hatch), fledging success (the probability of one or more offspring fledging), chick survival (the proportion of chicks that survive in a successful brood), and productivity. The survival category included post-fledging survival, sub-adult survival, and adult survival. The movement category comprised immigration and emigration rates. A demographic gap was defined as the lack of research output for one of the demographic measures for a particular species.

#### Threat status and research output

Vulnerable (VU: 14 species), Endangered (EN: 11 species), and Critically Endangered (CR: six species) were categorised as ‘threatened’; and Least Concern (LC: 128 species) and Near Threatened (NT: ten species) as ‘non-threatened’ (S2 Table). A research output was defined as the number of species, countries and demographic measures studied in each paper. For example, Krapu (2000) [33] studied five species in one country and measured nesting success (productivity demographic measure) for each of the five species. This paper produced five research outputs. If the study had taken place in two countries, then the research output would be ten. A chi-square analysis was used to determine if volume of research output differed between threatened and non-threatened species. Research output for threatened and non-threatened species was calculated for two time periods: 1977–1993; and 1994–2010. In 1994, the IUCN adopted the current threat categorisation procedure [19]. A Mann-Whitney U-test was used to determine whether there was a significantly greater increase (percentage difference between pre- and post-1994 publication rates) in research on threatened wildfowl species than on non-threatened species. The significance level (alpha) was set at *P* = 0.05 for all statistical tests.

#### A metric for directing future wildfowl demographic research

A research priority metric (RPM) was devised to reflect the importance of obtaining additional demographic information for each wildfowl species. The RPM was defined as

$$\text{RPM}=\left(1\right)\;{\text{RO}}_{\;\text{index}\;0-10}+\left(2\right)\;{\text{ER}}_{\;\text{index}\;2-10}+\left(3\right)\;{\text{CS}}_{\;\text{index}\;0-5}+\left(4\right)\;{\text{ROCS}}_{\;\text{index}\;0-5}$$

and accounts for: (1) the total research output (RO) for each wildfowl species, converted to a ranked index of 0–10. The 169 wildfowl species were divided into six groups based on amount of research output. A RO score of 10 was given to species with a research output of 0–1; 8 to species with a research output of 2–3; 6 to species with output of 4–6; 4 to species with output of 7–17; 2 to species with output of 18–31; and a score of 0 to species with output greater than 32. (2) The extinction risk (ER), based on the species’ Red List categories and defined as intervals of extinction risk (Least Concern = 1; Near Threatened = 2; Vulnerable = 3; Endangered = 4; and Critically Endangered = 5). ER weightings were based on a method similar to that used by Butchart et al. 2004 [34]. The index had equal increments across extinction risk categories, as it may be important to understand demographic rates of lesser-threatened species before they become seriously endangered. (3) The number of congeneric species (CS), converted to a ranked index of 0 to 5. A CS score of 5 was given to species with 0–5 congeneric species; 4 to species with 6–10 congenerics; 3 to species with 11–15; 2 to species with 16–20; 1 to species with 21–25; and a score of 0 was given to species with more than 26 congeneric species. (4) The research output of congeneric species (ROCS), was expressed as a ranked index 0–5, where 5 was given to species with 0–10 research outputs from congeneric species; 4 to species with 11–20; 3 to species with 21–30; 2 to species with 31–40; 1 to species with 41–50; and a score of 0 was given to species with more than 51 research outputs from congeneric species. Both CS and ROCS were used in the metric as it was deemed more valuable to have some data from many congeneric species compared with a lot of research output from just one congeneric species. Wildfowl species with a high RPM would exhibit a low research output, high threat status, and a small number of congeneric species with a low research output. Values of the metric will, of course alter over time, following changes in threat status and taxonomic revisions, but will be driven mainly by new research on the species itself and to a lesser extent, its congeners. We devised the metric as an additive algorithm to make it simple and repeatable, and to account for variation in the relative importance of each component. Metric and component values were calculated for all wildfowl species and are given in Supporting Information (S2 Table).

## Results

**Fig 2:**
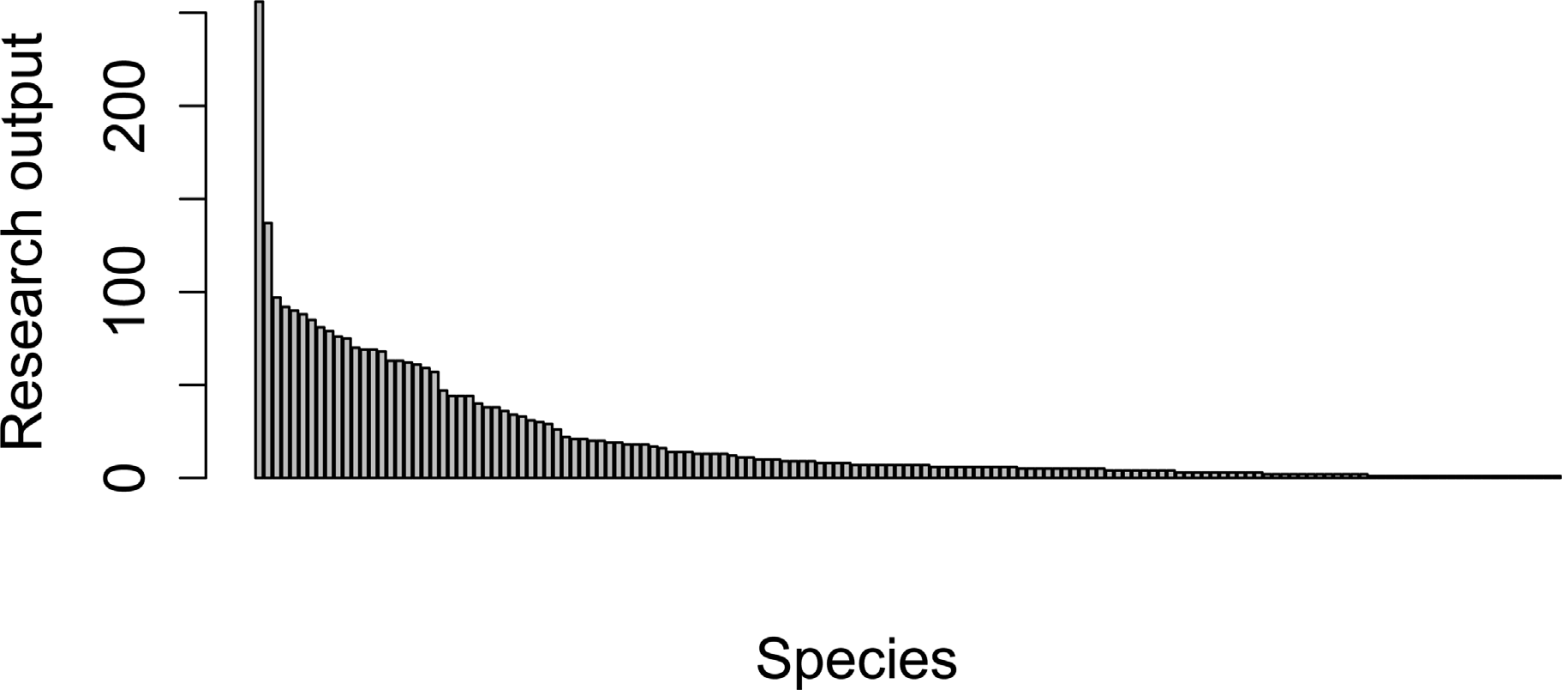
The number of demographic research outputs from 1911–2010 for wildfowl species, ranked by number of outputs.

The screened literature search resulted in 1,586 papers, yielding a total of 4,021 research outputs. Three journals contributed over a third of the publication total: Journal of Wildlife Management (19.3%); Wildfowl (10.6%); and The Auk (6.6%). Nearly 90% of wildfowl species had at least one demographic research output (Fig 2). Species found in North America and Europe were the subject of over 90% of the research outputs. Over half (55%) of the research output concerned just 15 species from seven genera. The most-studied species were Mallard (*Anas platyrhynchos*; 8.1% of output) and Northern Pintail (*Anas acuta*; 5.1%), and seven of the 15 most frequently studied species were from just three genera (*Anas*, *Aythya*, and *Anser*). There were 21 species with no demographic research output, and seven of these are globally threatened: the Critically Endangered Pink-headed Duck (*Rhodonessa caryophyllacea*) and Baer’s Pochard (*Aythya baeri*); the Endangered Campbell Teal (*Anas nesiotis*); and the Vulnerable White-headed Steamerduck (*Tachyeres leucocephalus*), Southern Pintail (*Anas eatoni*), Philippine Duck (*Anas luzonica*), and Swan Goose (*Anser cygnoides*).

**Fig 3:**
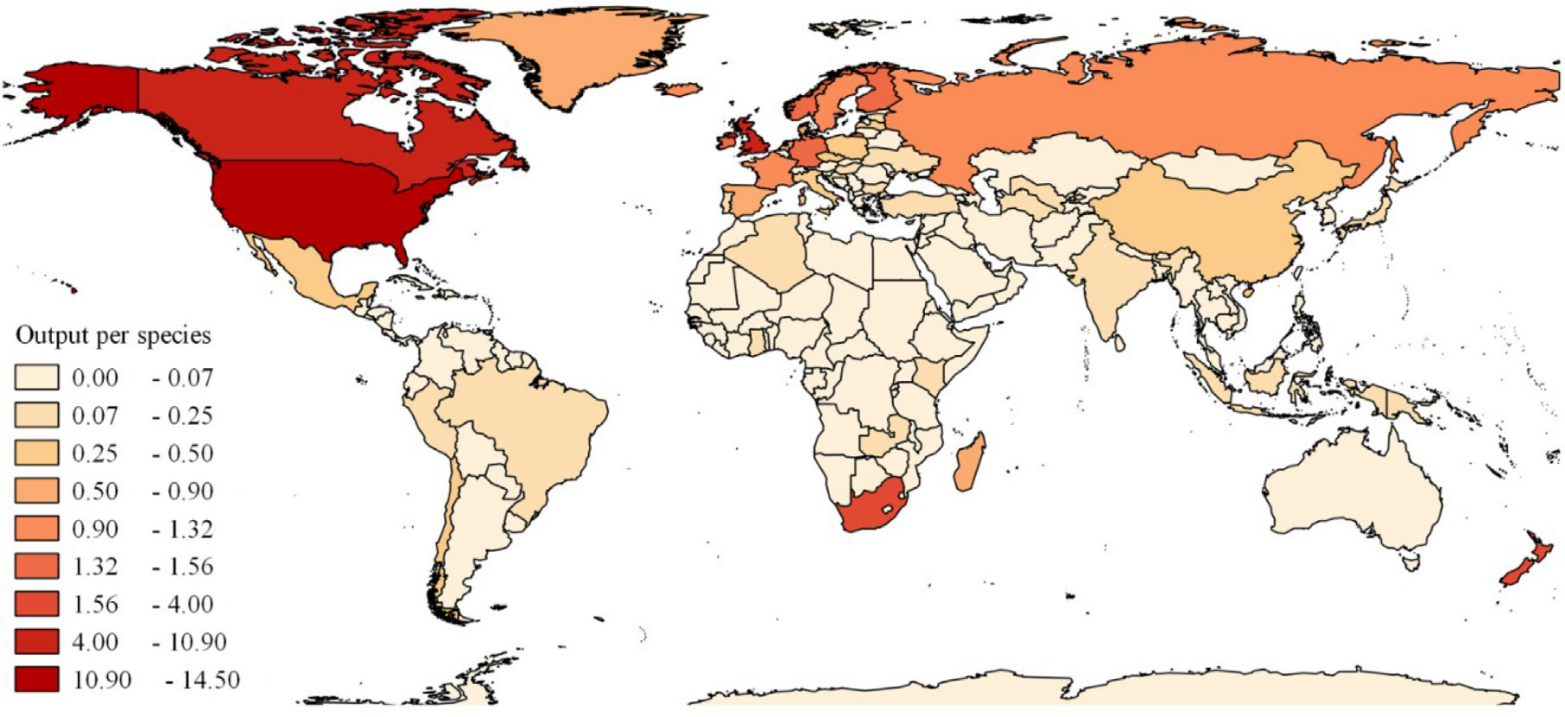
The number of demographic research outputs per wildfowl species for each country from 1911–2010.

All 75 wildfowl species occurring in North America and Europe had research output. Of the 21 species with no research output, 19 were from Asia or South America and two were from Australasia (Green Pygmy-goose *Nettapus pulchellus* and Campbell Teal; S2 Table). The United States, Canada, and the United Kingdom together produced >60% of the total research output (Fig 3). Of the ten highest ranked countries for number of research outputs, nine are classified by the World Bank as ‘high income’ countries (the tenth was ‘middle income’ South Africa). ‘High income’ countries contributed 90% of the research output while ‘lower income’ countries contributed only 0.1%. There was a significant, small positive effect of GNI on research output (β = +0.0010, *P* <0.001) and a non-significant, small positive trend for the number of wildfowl on research output (β = +0.035, *P* = 0.075).

**Fig 4:**
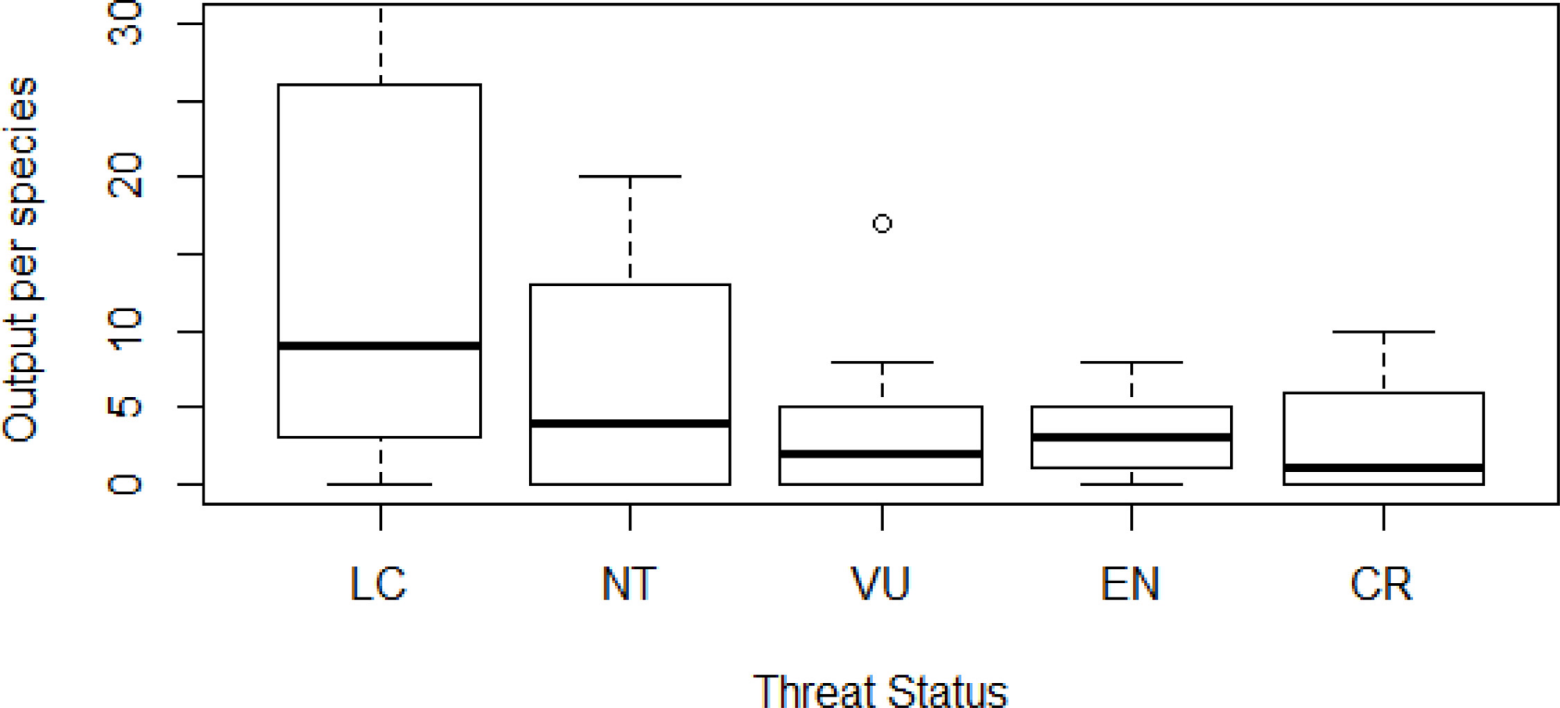
Boxplots showing median, upper and lower quartile, and 95% percentiles for number of demographic research outputs in species from different IUCN threat categories (1994–2010). LC = Least Concern; NT = Near Threatened; VU = Vulnerable; EN = Endangered; CR = Critically Endangered.

Least Concern species were the subject of significantly more research outputs than other threat categories (χ^2^ = 152, df = 4, *P* <0.001; Fig 4). Research output after the IUCN adopted the current threat categories in 1994 was significantly greater for threatened species (362%) than it was for non-threatened species (162%; Mann-Whitney U = 3090, *P* <0.001).

**Fig 5:**
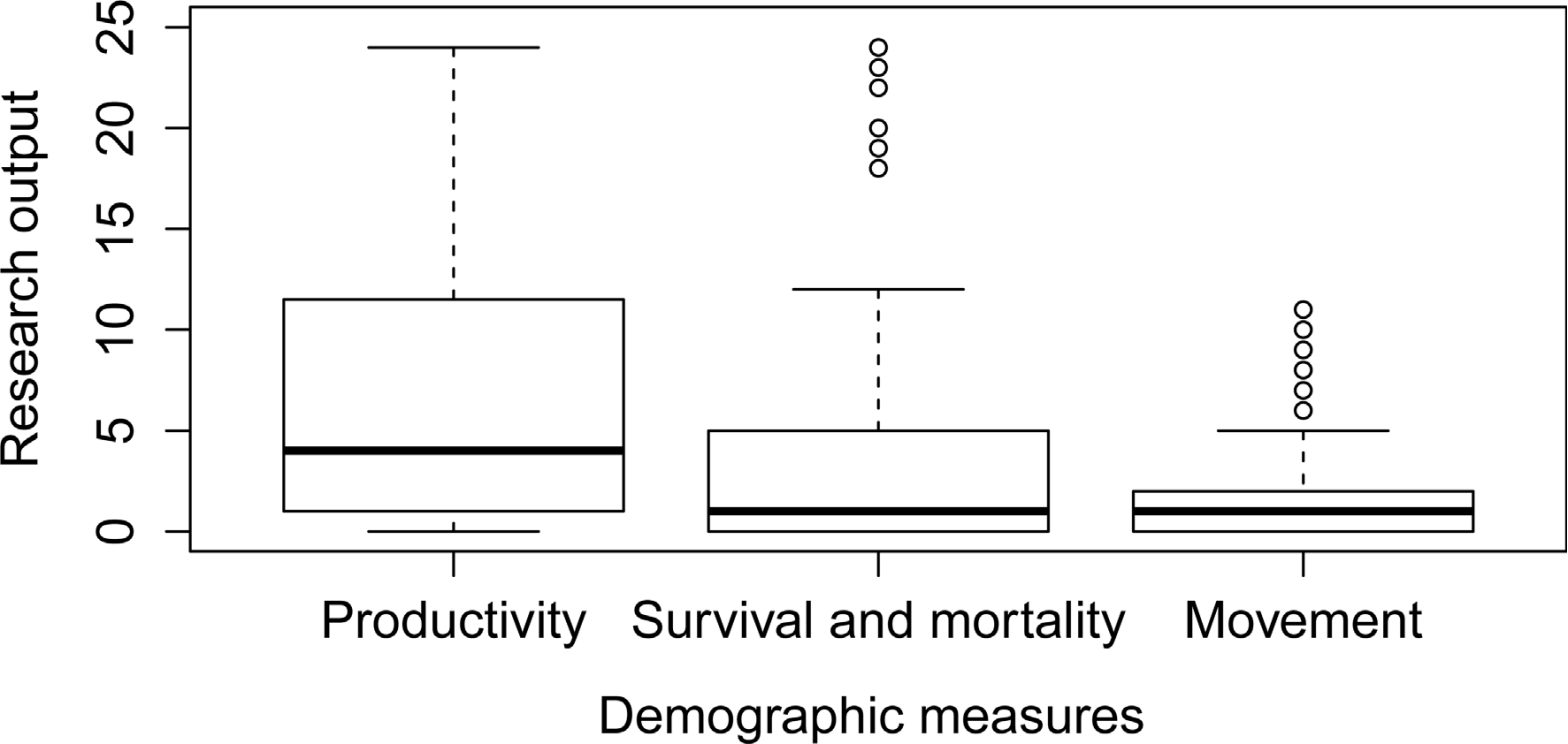
Distribution of demographic research output across three grouped demographic measures from 1911–2010.

In 2010, there were 164 demographic gaps in total. Measures of productivity were the most common demographic parameters studied for wildfowl (Fig 5) and dominated the research effort, with 52% of the total output. Survival estimates and movement data contributed 29% and 19% respectively.

Of the 25 species with the highest priority metric (RPM), 23 species are found outside Europe and North America and thirteen of the species are from monotypic genera (Table 1). Pink-headed Duck was the highest placed wildfowl species, and may actually be extinct in the wild [35]. Most species with very high RPM are highly threatened birds from the tropics (e.g., White-winged Duck *Cairina scutulata*, Blue-winged Goose *Cyanochen cyanopterus*, Crested Shelduck *Tadorna cristata*, White- headed Steamerduck). The exceptions are Marbled Teal (*Marmaronetta angustirostris*) and White-headed Duck (*Oxyura leucocephala*), which were included due to their threat status and low research output. The RPM metric was significantly positively correlated with all four component measures (r_smin_ = +0.32, *P*_max_ <0.005). Correlations between pairs of component measures were generally weaker, although RO was significantly correlated with ER (r_s_ = +0.34, *P* <0.005), CS (r_s_ = +0.45, *P* <0.005), and ROCS (r_s_ = +0.22, *P* <0.005). There was no correlation between ER and either ROCS (r_s_ = -0.02, *P* = 0.81), or CS (r_s_ = +0.06, *P* = 0.44). Notably, CS, the number of congeneric species, and ROCS, the research output for those congenerics, were uncorrelated (r_s_ = +0.03, *P* = 0.67).

**Table 1:** Summary of the global research priority metric (PRM) for the top 25 wildfowl species, ordered by the highest priority wildfowl species for future research.

Rank	Vernacular name	Scientific name	Research priority metric (RPM)	Demographic research output score (RO)	Threat score (ER)	Congeneric species score (CS)	Demographic research output of congeneric species score (ROCS)
1	Pink-headed Duck	*Rhodonessa caryophyllacea*	30	10 (0)ª	10 (CR)	5 (0)	5 (0)
2	White-winged Duck	*Asarcornis scutulata*	26	8 (3)	8 (EN)	5 (0)	5 (0)
3	Blue-winged Goose	*Cyanochen cyanoptera*	26	10 (1)	6 (VU)	5 (0)	5 (0)
4	White-headed Steamerduck	*Tachyeres leucocephalus*	25	10 (0)	6 (VU)	5 (3)	4 (15)
5	Crested Shelduck	*Tadorna cristata*	25	10 (1)	10 (CR)	5 (5)	0 (56)
6	Orinoco Goose	*Neochen jubata*	24	10 (0)	4 (NT)	5 (0)	5 (0)
7	Salvadori's Teal	*Salvadorina waigiuensis*	24	8 (2)	6 (VU)	5 (0)	5 (0)
8	Baer's Pochard	*Aythya baeri*	23	10 (0)	10 (CR)	3 (11)	0 (534)
9	Madagascar Pochard	*Aythya innotata*	23	10 (1)	10 (CR)	3 (11)	0 (533)
10	Brazilian Teal	*Amazonetta brasiliensis*	22	10 (1)	2 (LC)	5 (0)	5 (0)
11	Ringed Teal	*Callonetta leucophrys*	22	10 (1)	2 (LC)	5 (0)	5 (0)
12	Northern Screamer	*Chauna chavaria*	22	10 (0)	4 (NT)	5 (1)	5 (1)
13	Blue Duck	*Hymenolaimus malacorhynchos*	22	4 (7)	8 (EN)	5 (0)	5 (0)
14	Crested Duck	*Lophonetta specularioides*	22	10 (1)	2 (LC)	5 (1)	5 (0)
15	Marbled Teal	*Marmaronetta angustirostris*	22	6 (6)	6 (VU)	5 (0)	5 (0)
16	Brazilian Merganser	*Mergus octosetaceus*	22	6 (6)	10 (CR)	5 (3)	1 (48)
17	Scaly-sided Merganser	*Mergus squamatus*	22	8 (3)	8 (EN)	5 (3)	1 (51)
18	African Pygmy-goose	*Nettapus auritus*	22	10 (1)	2 (LC)	5 (2)	5 (7)
19	Green Pygmy-goose	*Nettapus pulchellus*	22	10 (0)	2 (LC)	5 (2)	5 (3)
20	White-headed Duck	*Oxyura leucocephala*	22	8 (3)	8 (EN)	5 (5)	1 (46)
21	Hartlaub's Duck	*Pteronetta hartlaubii*	22	10 (1)	2 (LC)	5 (0)	5 (0)
22	Radjah Shelduck	*Radjah radjah*	22	10 (0)	2 (LC)	5 (0)	5 (0)
23	American Comb Duck	Sarkidiornis sylvicola	22	10 (0)	2 (LC)	5 (1)	5 (4)
24	Baikal Teal	*Sibirionetta formosa*	22	10 (1)	2 (LC)	5 (0)	5 ()
25	Spectacled Duck	*Speculanas specularis*	22	4 (13)	4 (NT)	5 (0)	5 (0)

ªRaw data in parentheses.

## Discussion

Although 1,586 wildfowl research papers were published between 1911 and 2010, there remain significant gaps in knowledge of wildfowl demographics. Research has been intensely focused on a few common species, usually in ‘high income’ countries, leaving some of the world’s most threatened wildfowl species with little or no published demographic data to inform their conservation. Our research priority metric is intended to act as a tool to help redress this imbalance.

For many wildfowl species, the quantity and quality of demographic information is too limited to underpin precise conservation management. A high proportion of wildfowl species have very little information, with >60% of taxa having ten or fewer research outputs, and only 17% of threatened species having one or more output from all of the three demographic categories. Productivity measures are the most commonly captured, most likely due to the ease of collecting them [36]. Whilst there has been an increase in research output since 1994 for threatened species, demographic research output remains low (Fig 4). Whilst it is likely that some of the research imbalance is related to the difficulty of studying small populations [37], filling these research gaps in the world’s most seriously endangered wildfowl is a priority.

The IUCN Red List is an accepted tool for conservation priority setting [38], yet gaps in our knowledge of the demographic biology for particular species are known to be important [39], because this knowledge deficit reduces our capacity to develop an effective management strategy for threatened species [9]. It has been recognised that systematic planning of species conservation should integrate assessment of demographic knowledge gaps with threat status [40]. However, there have been few attempts in the literature to quantify gaps in knowledge for conservation purposes. Consideration has been given to research output and the number of range-restricted bird species for different areas [10], and, more generally, the EDGE prioritisation metric is a function of phylogenetic distinctiveness and threat status [41]. Further, RPM takes into consideration the value of information on related species [42]. This is valuable because the life histories of closely related species are more similar than distantly related species, and in the absence of demographic information, known rates for closely related species may provide surrogate demographic rates [43]. Having a body of demographic data on a threatened species is rarely going to be sufficient in itself, as specific data are required on that population at that given time [15]. However, demographic information from other populations or conspecifics can help to give context to the newly gathered data [13]. Evidence suggests that conservation research is correcting some of the biases, but progress is slow [8].

While several other papers have looked at general bias in conservation research [44, 45, 46], here, for the first time, we examine knowledge gaps in demographic research across a range of wildfowl species. The analyses demonstrate that research output is not randomly distributed according to geography, taxonomy, extinction risk, or demographic category, and that some of this bias is related to economic productivity in ‘low income’ countries. Some of the most under-studied species are the ones most in need of future protection. These include Critically Endangered species from low-income African and Asian countries/regions, which have declined rapidly due to habitat loss and direct exploitation (e.g. Baer’s Pochard; Madagascar Pochard *Aythya innotata*) [47]. Two Asian species, Crested Shelduck and Pink-headed Duck, may already be extinct in the wild but are still being sought [35].

This paper exposes the need to examine extinction risk alongside knowledge gaps for species-conservation setting approaches. The RPM developed in this paper could be applied across many taxonomic groups. Simple metrics, like RPM, are needed to set legal and conservation management targets, and could be a useful tool for organisations such as The Wildfowl & Wetlands Trust. Future conservation policy should routinely consider knowledge gaps by implementing metrics like RPM.

## Supporting Information

S1 Fig The number of wildfowl demographic research outputs per year from 1911–2010. (TIF)

S1 Table Search terms used for paper selection. (DOCX)

S2 Table Global research priority metric (RPM) for all wildfowl species, ordered by the highest priority wildfowl species for future research. (DOCX)

S3 Table PRISMA Checklist. (DOC)

## References

[1] Loreau M, Shahid N, Inchausti P, Bengtsson J, Grime JP, Hector A, et al Biodiversity and ecosystem functioning: current knowledge and future challenges. Science. 2001;294(5543): 804–808. 11679658 10.1126/science.1064088

[2] Butchart SHM, Stattersfield AJ, Collar NJ. How many bird extinctions have we prevented? Oryx. 2006;40(03): 266–278.

[3] BirdLife International. State of the world’s birds: indicators for our changing world Cambridge, UK: BirdLife International 2013;8: 20 Available: http://www.birdlife.org/datazone/sowb/SOWB2013.

[4] McCarthy MA, Thompson CJ, Hauser C, Burgman MA, Possingham HP, Moir ML, et al Resource allocation for efficient environmental management. Ecol Lett. 2010;13(10): 1280–1289. 10.1111/j.1461-0248.2010.01522.x 20718844

[5] Mace GM, Collar NJ, Gaston KJ, Hilton-Taylor C, Akçakaya HR, Leader-Williams NEJ, et al Quantification of extinction risk: IUCN's system for classifying threatened species. Conserv Biol. 2008;22(6): 1424–1442. 10.1111/j.1523-1739.2008.01044.x 18847444

[6] Hoffmann M, Brooks TM, da Fonseca GAB, Gascon C, Hawkins AFA, James RE, et al Conservation planning and the IUCN Red List. Endanger Species Res. 2008;6(2): 113–125.

[7] Schipper J, Chanson JS, Chiozza F, Cox NA, Hoffmann M, Katariya V, et al The status of the world's land and marine mammals: diversity, threat, and knowledge. Science. 2008;322(5899): 225–230. 10.1126/science.1165115 18845749

[8] Lawler JJ, Aukema JE, Grant JB, Halpern BS, Kareiva P, Nelson CR, et al Conservation science: a 20-year report card in a nutshell. Front Ecol Environ. 2006;4(9): 473–480.

[9] Pullin AS, Knight TM, Stone DA, Charman K. Do conservation managers use scientific evidence to support their decision-making? Biol Conserv. 2004;119(2): 245–252.

[10] Lima R, de Bird JP, Barlow J. Research effort allocation and the conservation of restricted-range island bird species. Biol Conserv. 2011;144(1): 627–632.

[11] Beissinger SR, Westphal MI. On the use of demographic models of population viability in endangered species management. J Wildlife Manage. 1998;1: 821–841.

[12] Coulson T, Gaillard J, Festa-Bianchet M. Decomposing the variation in population growth into contributions from multiple demographic rates. J Anim Ecol. 2005;74(4): 789–801.

[13] Sim IMW, Rebecca GW, Ludwig SC, Grant MC, Reid JM. Characterizing demographic variation and contributions to population growth rate in a declining population. J Anim Ecol. 2010;80(1): 159–170. 10.1111/j.1365-2656.2010.01750.x 20825517

[14] Bieber C, Ruf T. Population dynamics in wild boar Sus scrofa: ecology, elasticity of growth rate and implications for the management of pulsed resource consumers. J Appl Ecol. 2005;42(6): 1203–1213.

[15] Traill LW, Brook BW, Frankham RR, Bradshaw CJA. Pragmatic population viability targets in a rapidly changing world. Biol Conserv. 2010;143(1): 28–34.

[16] Lindenmayer DB, Clark TW, Lacy RC, Thomas VC. Population viability analysis as a tool in wildlife conservation policy: With reference to Australia. Environ Manage. 1993;17(6): 745–758.

[17] Kear J. Bird families of the world: Ducks, geese and swans Oxford: Oxford University Press; 2005.

[18] Nichols JD, Johnson FA, Williams BK. Managing North American waterfowl in the face of uncertainty. Annu Rev Ecol Syst. 1995;1: 177–199.

[19] IUCN. IUCN Red List of Threatened Species. Version 2014.3. IUCN 2014. 2014;12: 20 Available: http://www.iucnredlist.org.

[20] Long PR, Szekely T, Kershaw M, O’Connell M. Ecological factors and human threats both drive wildfowl population declines. Anim Conserv. 2007;10(2): 183–191.

[21] McCarthy DP, Donald PF, Scharlemann JP, Buchanan GM, Balmford A, Green JM, et al Financial costs of meeting global biodiversity conservation targets: current spending and unmet needs. Science. 2012;338(6109): 946–949. 10.1126/science.1229803 23065904

[22] James AN, Gaston KJ, Balmford A. Balancing the Earth's accounts. Nature. 1999;401(6751): 323–324. 16862091 10.1038/43774

[23] Gaston KJ. Global patterns in biodiversity. Nature. 2000;405(6783): 220–227. 10821282 10.1038/35012228

[24] Barber PH, Ablan-Lagman MCA, Berlinck RG, Cahyani D, Crandall ED, Ravago-Gotanco R, et al Advancing biodiversity research in developing countries: the need for changing paradigms. Bull Mar Sci. 2012;90(1): 187–210.

[25] Githiru M, King MW, Bauche P, Simon C, Boles J, Rindt C et al Should biodiversity offsets help finance underfunded Protected Areas? Biol Cons. 2015;191: 819–826.

[26] Moher D, Liberati A, Tetzlaff J, Altman DG. Preferred reporting items for systematic reviews and meta-analyses: the PRISMA statement. PLoS Med. 2009;6(7): e1000097 10.1371/journal.pmed.1000097 19621072 PMC2707599

[27] ISI Web of Knowledge. Suite of databases (List of databases that are part of the Web of Knowledge suite.). Thomson Reuters. 2012;2: 1 Available: http://thomsonreuters.com/web-of-knowledge/.

[28] Giles J. Science in the web age: Start your engines. Nature. 2005;438(7068): 554–555. 16319857 10.1038/438554a

[29] Whitehead AL, Edge KA, Smart AF, Hill GS, Willans MJ. Large scale predator control improves the productivity of a rare New Zealand riverine duck. Biol Conserv. 2008;141(11): 2784–2794.

[30] World Bank. World Development Indicators database. 2013;7: 23. Available: http://data.worldbank.org/data-catalog/world-development-indicator.

[31] Original S code by Becker RA, Allan R, Wilks R, version by Brownrigg R. Enhancements by Thomas P Minka and Alex Deckmyn. maps: Draw Geographical Maps. R package version 3.0.0–2. 2015. Available: http://CRAN.R-project.org/package=maps.

[32] Zuur A, Ieno EN, Walker N, Saveliev AA, Smith GM. Mixed effects models and extensions in ecology with R Springer Science & Business Media; 2009.

[33] Krapu GL. Temporal flexibility of reproduction in temperate-breeding dabbling ducks. Auk. 2000;117(3): 640–650.

[34] Butchart SHM, Stattersfield AJ, Bennun LA, Shutes SM, Akçakaya HR, Baillie JEM, et al Measuring global trends in the status of biodiversity: Red List Indices for birds. PLoS Biol. 2004;2(12): e383 15510230 10.1371/journal.pbio.0020383 PMC524254

[35] Tordoff AW, Appleton T, Eames IC. The historical and current status of Pink-headed Duck *Rhodonessa caryophyllacea* in Myanmar. Bird Conserv Int. 2008;18(01): 38–52.

[36] Skalski JR, Ryding KE, Millspaugh JJ. Primer on Wildlife Dynamics In: Wildlife Demography: analysis of sex, age, and count data. Academic Press; 2010 pp. 11–47.

[37] Barnes RFW. The problem of precision and trend detection posed by small elephant populations in West Africa. Afr J Ecol. 2002;40(2): 179–185.

[38] Schmeller DS, Bauch B, Gruber B, Juškaitis R, Budrys E, Babij V, et al Determination of conservation priorities in regions with multiple political jurisdictions. Biodivers Conserv. 2008;17(14): 3623–3630.

[39] Mace GM, Hudson EJ. Attitudes towards sustainability and extinction. Conserv Biol. 1999;13(2): 242–246.

[40] Margules CR, Pressey RL. Systematic conservation planning. Nature. 2000;405(6783): 243–253. 10821285 10.1038/35012251

[41] Isaac NJ, Turvey ST, Collen B, Waterman C, Baillie JE. Mammals on the EDGE: conservation priorities based on threat and phylogeny. PLoS ONE. 2007;2(3): e296 17375184 10.1371/journal.pone.0000296 PMC1808424

[42] Barker G. Phylogenetic diversity: a quantitative framework for measurement of priority and achievement in biodiversity conservation. Biol J Linn Soc. 2002;76(2): 165–194.

[43] Green AE. Applications of large scale studies of demographic rates to bird conservation. Bird Study. 1999;46(S1): S279–288.

[44] Clark J, May R. Taxonomic bias in conservation research. Science. 2002;297(5579): 191 12117005 10.1126/science.297.5579.191b

[45] Fisher R, Radford B. Global mismatch between research effort and conservation needs of tropical coral reefs. Conserv Lett. 2011;4(1): 64–72.

[46] Wilson KA, Underwood EC, Morrison SA, Klausmeyer KR, Murdoch WW, Reyers B, et al Conserving Biodiversity Efficiently: What to Do, Where, and When. (Mace Georgina M, Ed.). PLoS Biol. 2007;5(9): e223 17713985 10.1371/journal.pbio.0050223 PMC1950771

[47] Bamford AJ, Sam TS, Razafindrajao F, Robson H, Woolaver LG, René de Roland LA. The status and ecology of the last wild population of Madagascar Pochard Aythya innotata. Bird Cons Int. 2015;25(01): 97–110.

