# Supplementary figures and images for: Taxonomic and Geographic Bias in Conservation Biology Research: A Systematic Review of Wildfowl Demography Studies
Source: PLoS One. 2016 May 11;11(5):e0153908. doi: 10.1371/journal.pone.0153908 (PMC4864074; doi:10.1371/journal.pone.0153908)

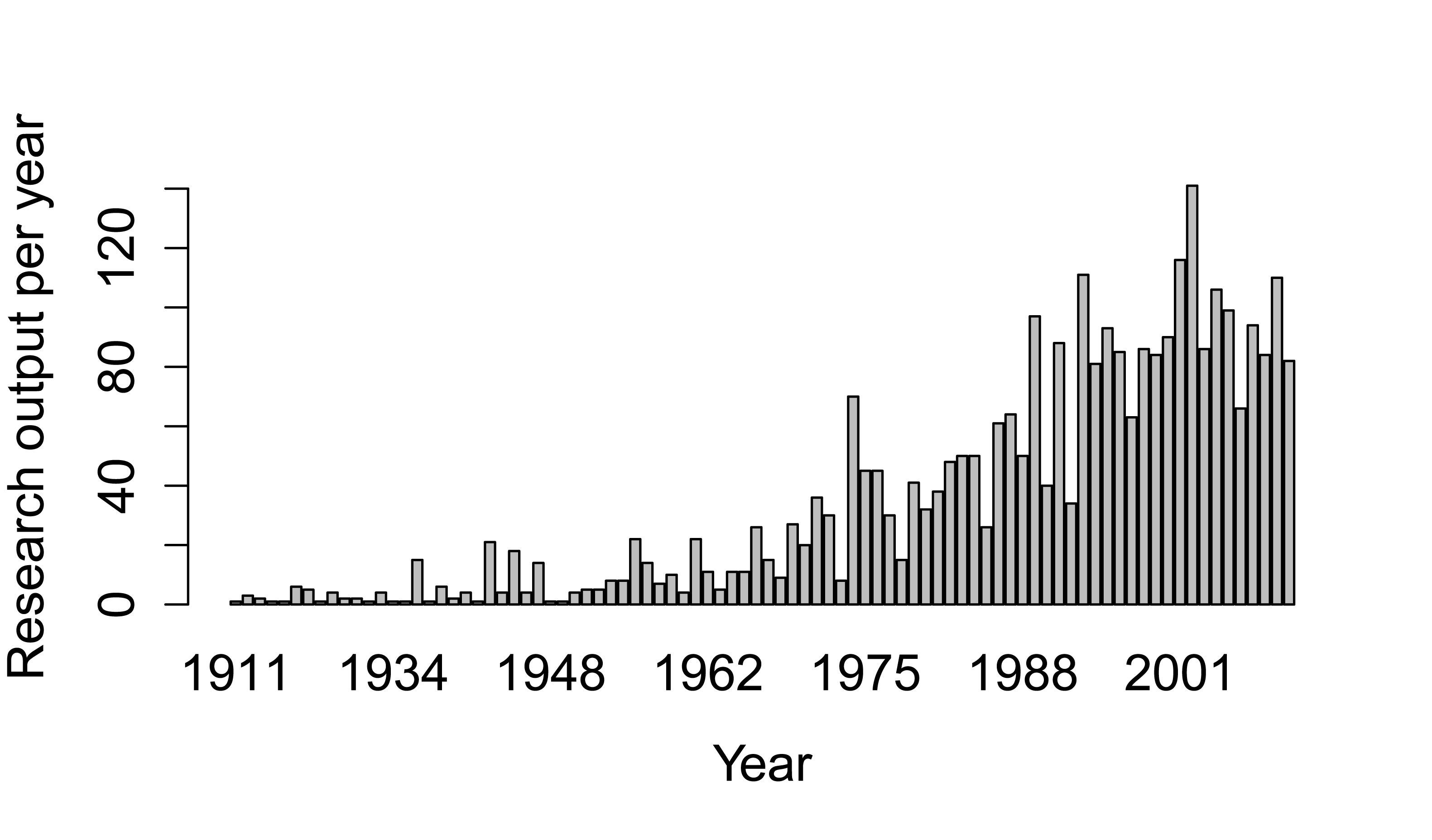

Supplement: S1 Fig — (TIF) [file pone.0153908.s001.tif]
