# Supplementary material for: Taxonomic and Geographic Bias in Conservation Biology Research: A Systematic Review of Wildfowl Demography Studies
Source: PLoS One. 2016 May 11;11(5):e0153908. doi: 10.1371/journal.pone.0153908 (PMC4864074; doi:10.1371/journal.pone.0153908)
Supplement: S1 Table — (DOCX) [file pone.0153908.s002.docx]

|  |
| --- |

**S1 Table. Search terms used for paper selection.**

| **Vernacular name** | **Scientific name** | **Historic and common name/s** |
| --- | --- | --- |
| Mandarin Duck | *Aix galericulata* | “Mandarin” |
| Wood Duck | *Aix sponsa* |  |
| Egyptian Goose | *Alopochen aegyptiaca* |  |
| Brazilian Teal | *Amazonetta brasiliensis* | “Brazilian Duck” |
| Northern Pintail | *Anas acuta* |  |
| Andaman Teal | *Anas albogularis* |  |
| Andean Teal | *Anas andium* |  |
| Auckland Teal | *Anas aucklandica* | “Auckland Islands Teal” |
| White-cheeked Pintail | *Anas bahamensis* | “Bahama Pintail”, “Summer Duck” |
| Madagascar Teal | *Anas bernieri* |  |
| Cape Teal | *Anas capensis* |  |
| Chestnut Teal | *Anas castanea* |  |
| Brown Teal | *Anas chlorotis* | “Pateke” |
| Common Teal | *Anas crecca* | “Green-winged Teal” |
| Southern Pintail | *Anas eatoni* | “Eaton's Pintail” |
| Red-billed Duck | *Anas erythrorhyncha* |  |
| Yellow-billed Teal | *Anas flavirostris* | “Merida Teal”, “Nominate” |
| Mottled Duck | *Anas fulvigula* | “Mottled Mallard” |
| Yellow-billed Pintail | *Anas georgica* |  |
| Sunda Teal | *Anas gibberifrons* |  |
| Grey Teal | *Anas gracilis* |  |
| Laysan Duck | *Anas laysanensis* | “Laysan Teal” |
| Philippine Duck | *Anas luzonica* |  |
| Meller's Duck | *Anas melleri* |  |
| Campbell Teal | *Anas nesiotis* |  |
| Mallard | *Anas platyrhynchos* |  |
| Indian Spot-billed Duck | *Anas poecilorhyncha* | “Western Spot-billed Duck” |
| American Black Duck | *Anas rubripes* |  |
| African Black Duck | *Anas sparsa* |  |
| Pacific Black Duck | *Anas supercilliosa* | “Grey Duck” |
| Yellow-billed Duck | *Anas undulata* |  |
| Hawaiian Duck | *Anas wyvilliana* |  |
| Chinese Spot-billed Duck | *Anas zonorhyncha* | “Koloa”, “Eastern Spot-billed Duck” |
| Horned Screamer | *Anhima cornuta* |  |
| Greater White-fronted Goose | *Anser albifrons* | “White-fronted Goose”, “Greater Whitefront” |
| Greylag Goose | *Anser anser* | “Graylag” |
| Pink-footed Goose | *Anser brachyrhynchus* | “Pinkfoot” |
| Snow Goose | *Anser caerulescens* | “Blue Goose” |
| Emperor Goose | *Anser canagica* |  |
| Swan Goose | *Anser cygnoid* |  |
| Lesser White-fronted Goose | *Anser erythropus* |  |
| Bean Goose | *Anser fabalis* |  |
| Bar-headed Goose | *Anser indicus* |  |
| Ross's Goose | *Anser rossii* |  |
| Magpie Goose | *Anseranas semipalmata* |  |
| White-winged Duck | *Asarcornis scutulata* | “White-winged Wood Duck” |
| Lesser Scaup | *Aythya affinis* |  |
| Redhead | *Aythya americana* |  |
| Hardhead | *Aythya australis* | “White-eyed Duck” |
| Baer's Pochard | *Aythya baeri* |  |
| Ring-necked Duck | *Aythya collaris* |  |
| Common Pochard | *Aythya ferina* | “Northern Pochard” |
| Tufted Duck | *Aythya fuligula* |  |
| Madagascar Pochard | *Aythya innotata* |  |
| Greater Scaup | *Aythya marila* |  |
| New Zealand Scaup | *Aythya novaeseelandiae* | “Black Teal” |
| Ferruginous Duck | *Aythya nyroca* | “Ferruginous Pochard”, “White-eyed Pochard” |
| Canvasback | *Aythya valisineria* |  |
| Musk Duck | *Biziura lobata* |  |
| Brent Goose | *Branta bernicla* | “Brant” |
| Canada Goose | *Branta canadensis* |  |
| Cackling Goose | *Branta hutchinsii* |  |
| Barnacle Goose | *Branta leucopsis* |  |
| Red-breasted Goose | *Branta ruficollis* |  |
| Hawaiian Goose | *Branta sandvicensis* | “Nene” |
| Bufflehead | *Bucephala albeola* |  |
| Common Goldeneye | *Bucephala clangula* |  |
| Barrow's Goldeneye | *Bucephala islandica* |  |
| Muscovy Duck | *Cairina moschata* |  |
| Ringed Teal | *Callonetta leucophrys* |  |
| Cape Barren Goose | *Cereopsis novaehollandiae* |  |
| Northern Screamer | *Chauna chavaria* | “Black-necked Screamer” |
| Southern Screamer | *Chauna torquata* | “Crested Screamer” |
| Maned Duck | *Chenonetta jubata* | “Australian Wood Duck”, “Maned Goose” |
| Kelp Goose | *Chloephaga hybrida* |  |
| Andean Goose | *Chloephaga melanoptera* |  |
| Upland Goose | *Chloephaga picta* | “Magellan Goose” |
| Ashy-headed Goose | *Chloephaga poliocephala* |  |
| Ruddy-headed Goose | *Chloephaga rubidiceps* |  |
| Long-tailed Duck | *Clangula hyemalis* | “Oldsquaw” |
| Coscoroba Swan | *Coscoroba coscoroba* | “Whistling Swan” |
| Blue-winged Goose | *Cyanochen cyanoptera* |  |
| Black Swan | *Cygnus atratus* |  |
| Trumpeter Swan | *Cygnus buccinator* |  |
| Tundra Swan | *Cygnus columbianus* | “Bewick’s Swan” |
| Whooper Swan | *Cygnus cygnus* |  |
| Black-necked Swan | *Cygnus melanocoryphus* |  |
| Mute Swan | *Cygnus olor* |  |
| West Indian Whistling-duck | *Dendrocygna arborea* | “Whistling Duck”, “Tree duck” |
| Wandering Whistling-duck | *Dendrocygna arcuata* | “Whistling Duck”, “Tree duck” |
| Black-bellied Whistling-duck | *Dendrocygna autumnalis* | “Whistling Duck”, “Tree duck” |
| Fulvous Whistling-duck | *Dendrocygna bicolor* | “Whistling Duck”, “Tree duck” |
| Plumed Whistling-duck | *Dendrocygna eytoni* | “Whistling Duck”, “Tree duck” |
| Spotted Whistling-duck | *Dendrocygna guttata* | “Whistling Duck”, “Tree duck” |
| Lesser Whistling-duck | *Dendrocygna javanica* | “Whistling Duck”, “Tree duck” |
| White-faced Whistling-duck | *Dendrocygna viduata* | “Whistling Duck”, “Tree duck” |
| Black-headed Duck | *Heteronetta atricapilla* |  |
| Harlequin Duck | *Histrionicus histrionicus* |  |
| Blue Duck | *Hymenolaimus malacorhynchos* | “Whio” |
| Hooded Merganser | *Lophodytes cucullatus* |  |
| Crested Duck | *Lophonetta specularioides* |  |
| Pink-eared Duck | *Malacorhynchus membranaceus* |  |
| Falcated Duck | *Mareca falcata* |  |
| Eurasian Wigeon | *Mareca penelope* | “Eurasian Widgeon” |
| American Wigeon | *Mareca americana* | “American Widgeon” |
| Chiloe Wigeon | *Mareca sibilatrix* | “Southern Wigeon” |
| Gadwall | *Mareca strepera* |  |
| Marbled Teal | *Marmaronetta angustirostris* | “Marbled Duck” |
| Black Scoter | *Melanitta americana* | “American Scoter” |
| White-winged Scoter | *Melanitta deglandi* |  |
| Velvet Scoter | *Melanitta fusca* |  |
| Common Scoter | *Melanitta nigra* |  |
| Surf Scoter | *Melanitta perspicillata* |  |
| Siberian Scoter | *Melanitta stejnegeri* | “Stejneger’s Scoter”, “Asian White-winged Scoter” |
| Torrent Duck | *Merganetta armata* |  |
| Smew | *Mergellus albellus* |  |
| Common Merganser | *Mergus merganser* |  |
| Brazilian Merganser | *Mergus octosetaceus* |  |
| Red-breasted Merganser | *Mergus serrator* |  |
| Scaly-sided Merganser | *Mergus squamatus* | “Chinese Merganser” |
| Orinoco Goose | *Neochen jubata* |  |
| Southern Pochard | *Netta erythrophthalma* |  |
| Rosy-billed Pochard | *Netta peposaca* |  |
| Red-crested Pochard | *Netta rufina* |  |
| African Pygmy-goose | *Nettapus auritus* |  |
| Cotton Pygmy-goose | *Nettapus coromandelianus* |  |
| Green Pygmy-goose | *Nettapus pulchellus* |  |
| Masked Duck | *Nomonyx dominicus* |  |
| Andean Duck | *Oxyura ferruginea* |  |
| Blue-billed Duck | *Oxyura australis* |  |
| Ruddy Duck | *Oxyura jamaicensis* |  |
| White-headed Duck | *Oxyura leucocephala* |  |
| Maccoa Duck | *Oxyura maccoa* |  |
| Lake Duck | *Oxyura vittata* |  |
| Spur-winged Goose | *Plectropterus gambensis* |  |
| Steller's Eider | *Polysticta stelleri* |  |
| Hartlaub's Duck | *Pteronetta hartlaubii* |  |
| Radjah Shelduck | *Radjah radjah* | “Raja Shelduck” |
| Pink-headed Duck | *Rhodonessa caryophyllacea* |  |
| Salvadori's Teal | *Salvadorina waigiuensis* |  |
| African Comb Duck | *Sarkidiornis melanotos* | “Knob-billed Duck” |
| American Comb Duck | *Sarkidiornis sylvicola* |  |
| Baikal Teal | *Sibirionetta formosa* |  |
| Spectacled Eider | *Somateria fischeri* |  |
| Common Eider | *Somateria mollissima* |  |
| King Eider | *Somateria spectabilis* |  |
| Northern Shoveler | *Spatula clypeata* |  |
| Cinnamon Teal | *Spatula cyanoptera* |  |
| Blue-winged Teal | *Spatula discors* |  |
| Hottentot Teal | *Spatula hottentota* |  |
| Red Shoveler | *Spatula platalea* | “Red Shoveller” |
| Puna Teal | *Spatula puna* |  |
| Garganey | *Spatula querquedula* |  |
| Australian Shoveler | *Spatula rhynchotis* | “New Zealand Shoveler” |
| Cape Shoveler | *Spatula smithii* | “Cape Shoveller” |
| Silver Teal | *Spatula versicolor* | “Versicolor Teal” |
| Spectacled Duck | *Speculanas specularis* | “Bronze-winged Duck” |
| Baikal Teal | *Sibirionetta formosa* | “Bimaculate Duck”, “Squawk Duck” |
| Freckled Duck | *Stictonetta naevosa* |  |
| Falkland Steamerduck | *Tachyeres brachypterus* |  |
| White-headed Steamerduck | *Tachyeres leucocephalus* | “Chubut” |
| Flying Steamerduck | *Tachyeres patachonicus* |  |
| Flightless Steamerduck | *Tachyeres pteneres* |  |
| South African Shelduck | *Tadorna cana* | “Cape Shelduck” |
| Crested Shelduck | *Tadorna cristata* | “Korean Crested Shelduck” |
| Ruddy Shelduck | *Tadorna ferruginea* |  |
| Common Shelduck | *Tadorna tadorna* |  |
| Australian Shelduck | *Tadorna tadornoides* |  |
| Paradise Shelduck | *Tadorna variegata* |  |
| White-backed Duck | *Thalassornis leuconotus* |  |
