# Supplementary material for: Taxonomic and Geographic Bias in Conservation Biology Research: A Systematic Review of Wildfowl Demography Studies
Source: PLoS One. 2016 May 11;11(5):e0153908. doi: 10.1371/journal.pone.0153908 (PMC4864074; doi:10.1371/journal.pone.0153908)
Supplement: S2 Table — (DOCX) [file pone.0153908.s003.docx]

|  |
| --- |

**S2 Table. Global research priority metric (PRM) for all wildfowl species, ordered by the highest priority wildfowl species for future research.**

| **Vernacular name** | **Scientific name** | **Research priority metric (RPM)** | **Demographic research output score (RO)** | **Threat score (ER)** | **Congeneric species score (CS)** | **Demographic research output of congeneric species score (ROCS)** |
| --- | --- | --- | --- | --- | --- | --- |
| Pink-headed Duck | *Rhodonessa caryophyllacea* | 30 | 10 (0)ª | 10 (CR) | 5 (0) | 5 (0) |
| White-winged Duck | *Asarcornis scutulata* | 26 | 8 (3) | 8 (EN) | 5 (0) | 5 (0) |
| Blue-winged Goose | *Cyanochen cyanoptera* | 26 | 10 (1) | 6 (VU) | 5 (0) | 5 (0) |
| White-headed Steamerduck | *Tachyeres leucocephalus* | 25 | 10 (0) | 6 (VU) | 5 (3) | 4 (15) |
| Crested Shelduck | *Tadorna cristata* | 25 | 10 (1) | 10 (CR) | 5 (5) | 0 (56) |
| Orinoco Goose | *Neochen jubata* | 24 | 10 (0) | 4 (NT) | 5 (0) | 5 (0) |
| Salvadori's Teal | *Salvadorina waigiuensis* | 24 | 8 (2) | 6 (VU) | 5 (0) | 5 (0) |
| Baer's Pochard | *Aythya baeri* | 23 | 10 (0) | 10 (CR) | 3 (11) | 0 (534) |
| Madagascar Pochard | *Aythya innotata* | 23 | 10 (1) | 10 (CR) | 3 (11) | 0 (533) |
| Brazilian Teal | *Amazonetta brasiliensis* | 22 | 10 (1) | 2 (LC) | 5 (0) | 5 (0) |
| Ringed Teal | *Callonetta leucophrys* | 22 | 10 (1) | 2 (LC) | 5 (0) | 5 (0) |
| Northern Screamer | *Chauna chavaria* | 22 | 10 (0) | 4 (NT) | 5 (1) | 5 (1) |
| Blue Duck | *Hymenolaimus malacorhynchos* | 22 | 4 (7) | 8 (EN) | 5 (0) | 5 (0) |
| Crested Duck | *Lophonetta specularioides* | 22 | 10 (1) | 2 (LC) | 5 (1) | 5 (0) |
| Marbled Teal | *Marmaronetta angustirostris* | 22 | 6 (6) | 6 (VU) | 5 (0) | 5 (0) |
| Brazilian Merganser | *Mergus octosetaceus* | 22 | 6 (6) | 10 (CR) | 5 (3) | 1 (48) |
| Scaly-sided Merganser | *Mergus squamatus* | 22 | 8 (3) | 8 (EN) | 5 (3) | 1 (51) |
| African Pygmy-goose | *Nettapus auritus* | 22 | 10 (1) | 2 (LC) | 5 (2) | 5 (7) |
| Green Pygmy-goose | *Nettapus pulchellus* | 22 | 10 (0) | 2 (LC) | 5 (2) | 5 (3) |
| White-headed Duck | *Oxyura leucocephala* | 22 | 8 (3) | 8 (EN) | 5 (5) | 1 (46) |
| Hartlaub's Duck | *Pteronetta hartlaubii* | 22 | 10 (1) | 2 (LC) | 5 (0) | 5 (0) |
| Radjah Shelduck | *Radjah radjah* | 22 | 10 (0) | 2 (LC) | 5 (0) | 5 (0) |
| American Comb Duck | *Sarkidiornis sylvicola* | 22 | 10 (0) | 2 (LC) | 5 (1) | 5 (4) |
| Baikal Teal | *Sibirionetta formosa* | 22 | 10 (1) | 2 (LC) | 5 (0) | 5 () |
| Spectacled Duck | *Speculanas specularis* | 22 | 4 (13) | 4 (NT) | 5 (0) | 5 (0) |
| Red-breasted Goose | *Branta ruficollis* | 21 | 8 (2) | 8 (EN) | 5 (5) | 0 (211) |
| Kelp Goose | *Chloephaga hybrida* | 21 | 10 (1) | 2 (LC) | 5 (4) | 4 (20) |
| Andean Goose | *Chloephaga melanoptera* | 21 | 10 (0) | 2 (LC) | 5 (4) | 4 (21) |
| Falkland Steamerduck | *Tachyeres brachypterus* | 21 | 10 (1) | 2 (LC) | 5 (3) | 4 (14) |
| Horned Screamer | *Anhima cornuta* | 20 | 8 (2) | 2 (LC) | 5 (0) | 5 (0) |
| Swan Goose | *Anser cygnoid* | 20 | 10 (0) | 6 (VU) | 4 (9) | 0 (451) |
| Southern Screamer | *Chauna torquata* | 20 | 8 (1) | 2 (LC) | 5 (1) | 5 (0) |
| Long-tailed Duck | *Clangula hyemalis* | 20 | 4 (17) | 6 (VU) | 5 (0) | 5 (0) |
| Black-headed Duck | *Heteronetta atricapilla* | 20 | 8 (3) | 2 (LC) | 5 (0) | 5 (0) |
| Smew | *Mergellus albellus* | 20 | 8 (2) | 2 (LC) | 5 (0) | 5 (0) |
| Cotton Pygmy-goose | *Nettapus coromandelianus* | 20 | 8 (3) | 2 (LC) | 5 (2) | 5 (0) |
| Masked Duck | *Nomonyx dominicus* | 20 | 8 (3) | 2 (LC) | 5 (0) | 5 (0) |
| Steller's Eider | *Polysticta stelleri* | 20 | 4 (7) | 6 (VU) | 5 (0) | 5 (0) |
| West Indian Whistling-duck | *Dendrocygna arborea* | 19 | 8 (3) | 6 (VU) | 4 (7) | 1 (44) |
| Falcated Duck | *Mareca falcata* | 19 | 10 (0) | 4 (NT) | 5 (4) | 0 (340) |
| Madagascar Teal | *Anas bernieri* | 18 | 10 (1) | 8 (EN) | 0 (28) | 0 (921) |
| Meller's Duck | *Anas melleri* | 18 | 10 (1) | 8 (EN) | 0 (28) | 0 (921) |
| Campbell Teal | *Anas nesiotis* | 18 | 10 (0) | 8 (EN) | 0 (28) | 0 (922) |
| Hawaiian Duck | *Anas wyvilliana* | 18 | 10 (1) | 8 (EN) | 0 (28) | 0 (921) |
| Cape Barren Goose | *Cereopsis novaehollandiae* | 18 | 6 (5) | 2 (LC) | 5 (0) | 5 (0) |
| Maned Duck | *Chenonetta jubata* | 18 | 6 (6) | 2 (LC) | 5 (0) | 5 (0) |
| Pink-eared Duck | *Malacorhynchus membranaceus* | 18 | 6 (5) | 2 (LC) | 5 (0) | 5 (0) |
| Blue-billed Duck | *Oxyura australis* | 18 | 8 (3) | 4 (NT) | 5 (4) | 1 (46) |
| Maccoa Duck | *Oxyura maccoa* | 18 | 8 (3) | 4 (NT) | 5 (5) | 1 (46) |
| Lake Duck | *Oxyura vittata* | 18 | 10 (0) | 2 (LC) | 5 (5) | 1 (49) |
| African Comb Duck | *Sarkidiornis melanotos* | 18 | 6 (4) | 2 (LC) | 5 (1) | 5 (10) |
| White-backed Duck | *Thalassornis leuconotus* | 18 | 6 (4) | 2 (LC) | 5 (5) | 5 (0) |
| Egyptian Goose | *Alopochen aegyptiaca* | 17 | 4 (15) | 2 (LC) | 5 (0) | 5 (0) |
| Hawaiian Goose | *Branta sandvicensis* | 17 | 6 (4) | 6 (VU) | 5 (5) | 0 (209) |
| Ashy-headed Goose | *Chloephaga poliocephala* | 17 | 6 (4) | 2 (LC) | 5 (4) | 4 (17) |
| Ruddy-headed Goose | *Chloephaga rubidiceps* | 17 | 6 (5) | 2 (LC) | 5 (4) | 4 (16) |
| Black-necked Swan | *Cygnus melanocoryphus* | 17 | 10 (7) | 2 (LC) | 5 (5) | 0 (266) |
| Plumed Whistling-duck | *Dendrocygna eytoni* | 17 | 10 (1) | 2 (LC) | 4 (7) | 1 (46) |
| Spotted Whistling-duck | *Dendrocygna guttata* | 17 | 10 (0) | 2 (LC) | 4 (7) | 1 (47) |
| Lesser Whistling-duck | *Dendrocygna javanica* | 17 | 10 (0) | 2 (LC) | 4 (7) | 1 (47) |
| Velvet Scoter | *Melanitta fusca* | 17 | 4 (8) | 8 (EN) | 5 (5) | 0 (55) |
| Siberian Scoter | *Melanitta stejnegeri* | 17 | 10 (0) | 2 (LC) | 5 (5) | 0 (63) |
| Ruddy Shelduck | *Tadorna ferruginea* | 17 | 10 (1) | 2 (LC) | 5 (5) | 0 (56) |
| Andaman Teal | *Anas albogularis* | 16 | 10 (1) | 6 (VU) | 0 (28) | 0 (921) |
| Southern Pintail | *Anas eatoni* | 16 | 10 (0) | 6 (VU) | 0 (28) | 0 (922) |
| Philippine Duck | *Anas luzonica* | 16 | 10 (0) | 6 (VU) | 0 (28) | 0 (922) |
| Magpie Goose | *Anseranas semipalmata* | 16 | 4 (13) | 2 (LC) | 5 (0) | 5 (0) |
| Musk Duck | *Biziura lobata* | 16 | 4 (7) | 2 (LC) | 5 (0) | 5 (0) |
| Muscovy Duck | *Cairina moschata* | 16 | 6 (5) | 2 (LC) | 5 (0) | 5 (0) |
| Upland Goose | *Chloephaga picta* | 16 | 4 (11) | 2 (LC) | 5 (4) | 5 (10) |
| Coscoroba Swan | *Coscoroba coscoroba* | 16 | 4 (10) | 2 (LC) | 5 (0) | 5 (0) |
| Hooded Merganser | *Lophodytes cucullatus* | 16 | 4 (17) | 2 (LC) | 5 (1) | 5 (0) |
| Torrent Duck | *Merganetta armata* | 16 | 4 (8) | 2 (LC) | 5 (0) | 5 (0) |
| Andean Duck | *Oxyura ferruginea* | 16 | 8 (2) | 2 (LC) | 5 (5) | 1 (47) |
| Spur-winged Goose | *Plectropterus gambensis* | 16 | 4 (7) | 2 (LC) | 5 (0) | 5 (0) |
| Freckled Duck | *Stictonetta naevosa* | 16 | 4 (9) | 2 (LC) | 5 (0) | 5 (0) |
| Flying Steamerduck | *Tachyeres patachonicus* | 16 | 4 (7) | 2 (LC) | 5 (3) | 5 (8) |
| Magellanic Steamerduck | *Tachyeres pteneres* | 16 | 4 (7) | 2 (LC) | 5 (3) | 5 (8) |
| Red Shoveler | *Spatula platalea* | 16 | 10 (5) | 2 (LC) | 4 (9) | 0 (378) |
| Puna Teal | *Spatula puna* | 16 | 10 (0) | 2 (LC) | 4 (9) | 0 (383) |
| Wandering Whistling-duck | *Dendrocygna arcuata* | 15 | 8 (3) | 2 (LC) | 4 (7) | 1 (44) |
| Chiloe Wigeon | *Mareca sibilatrix* | 15 | 8 (2) | 2 (LC) | 5 (4) | 0 (338) |
| Rosy-billed Pochard | *Netta peposaca* | 15 | 8 (2) | 2 (LC) | 5 (2) | 0 (124) |
| New Zealand Scaup | *Aythya novaeseelandiae* | 15 | 10 (1) | 2 (LC) | 3 (11) | 0 (533) |
| Laysan Duck | *Anas laysanensis* | 14 | 4 (10) | 10 (CR) | 0 (28) | 0 (912) |
| Lesser White-fronted Goose | *Anser erythropus* | 14 | 4 (8) | 6 (VU) | 4 (9) | 0 (443) |
| Bean Goose | *Anser fabalis* | 14 | 4 (7) | 2 (LC) | 4 (9) | 0 (444) |
| Ross's Goose | *Anser rossii* | 14 | 4 (14) | 2 (LC) | 4 (9) | 0 (437) |
| Harlequin Duck | *Histrionicus histrionicus* | 14 | 2 (26) | 2 (LC) | 5 (0) | 5 (0) |
| South African Shelduck | *Tadorna cana* | 14 | 6 (10) | 2 (LC) | 5 (5) | 1 (47) |
| Silver Teal | *Spatula versicolor* | 14 | 8 (2) | 2 (LC) | 4 (9) | 0 (381) |
| Cackling Goose | *Branta hutchinsii* | 13 | 6 (4) | 2 (LC) | 5 (5) | 0 (209) |
| Common Scoter | *Melanitta nigra* | 13 | 6 (6) | 2 (LC) | 5 (5) | 0 (57) |
| Paradise Shelduck | *Tadorna variegata* | 13 | 4 (17) | 2 (LC) | 5 (5) | 2 (40) |
| Ferruginous Duck | *Aythya nyroca* | 13 | 6 (5) | 4 (NT) | 3 (11) | 0 (529) |
| Wood Duck | *Aix sponsa* | 12 | 0 (78) | 2 (LC) | 5 (1) | 5 (10) |
| Auckland Teal | *Anas aucklandica* | 12 | 6 (4) | 6 (VU) | 0 (28) | 0 (918) |
| Brown Teal | *Anas chlorotis* | 12 | 4 (7) | 8 (EN) | 0 (28) | 0 (915) |
| Yellow-billed Teal | *Anas flavirostris* | 12 | 10 (1) | 2 (LC) | 0 (28) | 0 (921) |
| Indian Spot-billed Duck | *Anas poecilorhyncha* | 12 | 10 (0) | 2 (LC) | 0 (28) | 0 (922) |
| Chinese Spot-billed Duck | *Anas zonorhyncha* | 12 | 10 (0) | 2 (LC) | 0 (28) | 0 (922) |
| Emperor Goose | *Anser canagicus* | 12 | 4 (14) | 4 (NT) | 4 (9) | 0 (437) |
| Bar-headed Goose | *Anser indicus* | 12 | 6 (4) | 2 (LC) | 4 (9) | 0 (447) |
| Fulvous Whistling-duck | *Dendrocygna bicolor* | 12 | 4 (12) | 2 (LC) | 4 (7) | 2 (35) |
| White-faced Whistling-duck | *Dendrocygna viduata* | 12 | 4 (8) | 2 (LC) | 4 (7) | 2 (39) |
| Black Scoter | *Melanitta americana* | 12 | 2 (20) | 4 (NT) | 5 (5) | 1 (43) |
| Red-crested Pochard | *Netta rufina* | 12 | 0 (116) | 2 (LC) | 5 (2) | 5 (10 ) |
| Australian Shelduck | *Tadorna tadornoides* | 12 | 4 (7) | 2 (LC) | 5 (5) | 1 (50) |
| Hottentot Teal | *Spatula hottentota* | 12 | 6 (6) | 2 (LC) | 4 (9) | 0 (377) |
| Mandarin Duck | *Aix galericulata* | 11 | 4 (10) | 2 (LC) | 5 (1) | 0 (78) |
| Bufflehead | *Bucephala albeola* | 11 | 4 (10) | 2 (LC) | 5 (2) | 0 (76) |
| Barrow's Goldeneye | *Bucephala islandica* | 11 | 4 (16) | 2 (LC) | 5 (2) | 0 (70) |
| Trumpeter Swan | *Cygnus buccinator* | 11 | 4 (16) | 2 (LC) | 5 (5) | 0 (257) |
| Black-bellied Whistling-duck | *Dendrocygna autumnalis* | 11 | 2 (20) | 2 (LC) | 4 (7) | 3 (27) |
| Surf Scoter | *Melanitta perspicillata* | 11 | 4 (9) | 2 (LC) | 5 (5) | 0 (54) |
| Goosander | *Mergus merganser* | 11 | 2 (19) | 2 (LC) | 5 (3) | 2 (35) |
| Red-breasted Merganser | *Mergus serrator* | 11 | 2 (26) | 2 (LC) | 5 (3) | 2 (28) |
| Southern Pochard | *Netta erythrophthalma* | 11 | 4 (8) | 2 (LC) | 5 (2) | 0 (118) |
| Ruddy Duck | *Oxyura jamaicensis* | 11 | 0 (38) | 2 (LC) | 5 (5) | 4 (11) |
| Spectacled Eider | *Somateria fischeri* | 11 | 4 (14) | 2 (LC) | 5 (2) | 0 (114) |
| Common Shelduck | *Tadorna tadorna* | 11 | 2 (21) | 2 (LC) | 5 (5) | 2 (36) |
| Yellow-billed Pintail | *Anas georgica* | 10 | 6 (6) | 2 (LC) | 0 (28) | 0 (916) |
| Common Goldeneye | *Bucephala clangula* | 10 | 0 (60) | 2 (LC) | 5 (2) | 3 (26) |
| White-winged Scoter | *Melanitta deglandi* | 10 | 2 (20) | 2 (LC) | 5 (5) | 1 (43) |
| Australian Shoveler | *Spatula rhynchotis* | 10 | 4 (9) | 2 (LC) | 4 (9) | 0 (374) |
| Cape Shoveler | *Spatula smithii* | 10 | 4 (11) | 2 (LC) | 4 (9) | 0 (372) |
| Black Swan | *Cygnus atratus* | 9 | 2 (18) | 2 (LC) | 5 (5) | 0 (255) |
| American Wigeon | *Mareca americana* | 9 | 2 (37) | 2 (LC) | 5 (4) | 0 (303) |
| Common Eider | *Somateria mollissima* | 9 | 0 (92) | 2 (LC) | 5 (2) | 2 (36) |
| King Eider | *Somateria spectabilis* | 9 | 2 (22) | 2 (LC) | 5 (2) | 0 (106) |
| White-cheeked Pintail | *Anas bahamensis* | 8 | 6 (4) | 2 (LC) | 0 (28) | 0 (918) |
| Chestnut Teal | *Anas castanea* | 8 | 6 (6) | 2 (LC) | 0 (28) | 0 (916) |
| Sunda Teal | *Anas gibberifrons* | 8 | 6 (4) | 2 (LC) | 0 (28) | 0 (918) |
| African Black Duck | *Anas sparsa* | 8 | 6 (6) | 2 (LC) | 0 (28) | 0 (916) |
| Cinnamon Teal | *Spatula cyanoptera* | 8 | 2 (18) | 2 (LC) | 4 (9) | 0 (365) |
| Brent Goose | *Branta bernicla* | 7 | 0 (73) | 2 (LC) | 5 (5) | 0 (140) |
| Canada Goose | *Branta canadensis* | 7 | 0 (95) | 2 (LC) | 5 (5) | 0 (118) |
| Barnacle Goose | *Branta leucopsis* | 7 | 0 (35) | 2 (LC) | 5 (5) | 0 (178) |
| Tundra Swan | *Cygnus columbianus* | 7 | 0 (32) | 2 (LC) | 5 (5) | 0 (241) |
| Whooper Swan | *Cygnus cygnus* | 7 | 0 (43) | 2 (LC) | 5 (5) | 0 (230) |
| Mute Swan | *Cygnus olor* | 7 | 0 (157) | 2 (LC) | 5 (5) | 0 (116) |
| Eurasian Wigeon | *Mareca penelope* | 7 | 0 (132) | 2 (LC) | 5 (4) | 0 (208) |
| Gadwall | *Mareca strepera* | 7 | 0 (169) | 2 (LC) | 5 (4) | 0 (171) |
| Hardhead | *Aythya australis* | 7 | 4 (9) | 2 (LC) | 3 (11) | 0 (525) |
| Ring-necked Duck | *Aythya collaris* | 7 | 2 (26) | 2 (LC) | 3 (11) | 0 (508) |
| Cape Teal | *Anas capensis* | 6 | 4 (10) | 2 (LC) | 0 (28) | 0 (912) |
| Red-billed Teal | *Anas erythrorhyncha* | 6 | 4 (10) | 2 (LC) | 0 (28) | 0 (912) |
| Pacific Black Duck | *Anas superciliosa* | 6 | 4 (9) | 2 (LC) | 0 (28) | 0 (913) |
| Yellow-billed Duck | *Anas undulata* | 6 | 4 (58) | 2 (LC) | 0 (28) | 0 (864) |
| Greater White-fronted Goose | *Anser albifrons* | 6 | 0 (141) | 2 (LC) | 4 (9) | 0 (310) |
| Greylag Goose | *Anser anser* | 6 | 0 (136) | 2 (LC) | 4 (9) | 0 (315) |
| Pink-footed Goose | *Anser brachyrhynchus* | 6 | 0 (43) | 2 (LC) | 4 (9) | 0 (408) |
| Snow Goose | *Anser caerulescens* | 6 | 0 (84) | 2 (LC) | 4 (9) | 0 (315) |
| Northern Shoveler | *Spatula clypeata* | 6 | 0 (168) | 2 (LC) | 4 (9) | 0 (215) |
| Blue-winged Teal | *Spatula discors* | 6 | 0 (53) | 2 (LC) | 4 (9) | 0 (330) |
| Garganey | *Spatula querquedula* | 6 | 0 (111) | 2 (LC) | 4 (9) | 0 (272) |
| Canvasback | *Aythya valisineria* | 5 | 0 (40) | 2 (LC) | 3 (11) | 0 (494) |
| Lesser Scaup | *Aythya affinis* | 5 | 0 (61) | 2 (LC) | 3 (11) | 0 (473) |
| Redhead | *Aythya americana* | 5 | 0 (61) | 2 (LC) | 3 (11) | 0 (473) |
| Common Pochard | *Aythya ferina* | 5 | 0 (146) | 2 (LC) | 3 (11) | 0 (388) |
| Tufted Duck | *Aythya fuligula* | 5 | 0 (152) | 2 (LC) | 3 (11) | 0 (382) |
| Greater Scaup | *Aythya marila* | 5 | 0 (32) | 2 (LC) | 3 (11) | 0 (502) |
| Mottled Duck | *Anas fulvigula* | 4 | 2 (15) | 2 (LC) | 0 (28) | 0 (907) |
| Grey Teal | *Anas gracilis* | 4 | 2 (19) | 2 (LC) | 0 (28) | 0 (903) |
| Northern Pintail | *Anas acuta* | 2 | 0 (207) | 2 (LC) | 0 (34) | 0 (715) |
| Common Teal | *Anas crecca* | 2 | 0 (162) | 2 (LC) | 0 (28) | 0 (720) |
| Mallard | *Anas platyrhynchos* | 2 | 0 (325) | 2 (LC) | 0 (28) | 0 (597) |
| American Black Duck | *Anas rubripes* | 2 | 0 (53) | 2 (LC) | 0 (28) | 0 (869) |

ªRaw data in parenthesis
